# Sensory Processing Difficulties in Youths With Disruptive Mood Dysregulation Disorder

**DOI:** 10.3389/fpsyt.2020.00164

**Published:** 2020-03-23

**Authors:** Xavier Benarous, Véronique Bury, Hélène Lahaye, Lyne Desrosiers, David Cohen, Jean Marc Guilé

**Affiliations:** ^1^ Department of Child and Adolescent Psychopathology, Amiens University Hospital, Amiens, France; ^2^ Department of Child and Adolescent Psychiatry, Pitié-Salpêtrière Hospital, Paris, France; ^3^ INSERM Unit U1105 Research Group for Analysis of the Multimodal Cerebral Function, University of Picardy Jules Verne (UPJV), Amiens, France; ^4^ Département d’ergothérapie, Trois-Rivières, Université du Québec à Trois-Rivières, Québec, Canada; ^5^ Centre de recherche de l’Institut Universitaire Jeunes en difficulté, Québec, Québec, Canada; ^6^ CNRS UMR 7222, Institute for Intelligent Systems and Robotics, Paris, France; ^7^ Department of Psychiatry, McGill University, Montreal, QC, Canada

**Keywords:** sensory processing difficulties, disruptive mood dysregulation disorder, chronic irritability, mood dysregulation, sensory over-responsivity, sensory modulation

## Abstract

Difficulty modulating sensory information has been described in children with developmental disorders. However, the relation of sensory processing difficulties (SPD) to emotional regulation problems remains poorly understood. The aim of this study is to determine the rate and patterns of SPD in youth with disruptive mood dysregulation disorder (DMDD). Participants were DMDD patients aged 6–16 years presenting at a university hospital in outpatient or inpatient facilities (n = 30). For each participant, the parent-reported Sensory Profile, the Affective Lability Scale, the Beck Depression Inventory-Second Edition, the Child Behavior Checklist/4–18, and the Kiddie Schedule for Affective Disorders and Schizophrenia for School-Age Children–Present and Lifetime Version were completed. The scores of the Sensory Profile of the DMDD youths were compared to those obtained in a clinical control group and to the manual scores for same-age typically developing youths. SPD were reported in 53% of the subjects in the DMDD group compared to 33% in the clinical control group (*p* = 0.405). Youths with DMDD showed a significant difference on almost all items of the Sensory Profile compared to typically developing youth. The Sensory Profile was found to discriminate best between the participants with DMDD and those in the clinical control group with regard to the category “Behavioral outcomes of sensory processing” and the factor “Fine motor/perceptual behavior.” All types of sensory processing patterns were reported in the DMDD youths: sensation avoiding (40%), low registration (27%), sensory sensitivity (20%), and sensation seeking (10%). As a group, youths with DMDD have significantly more SPD when compared to typical youths. Therefore, SPD could be an important factor to consider in youths with DMDD when providing comprehensive assessment and therapeutic interventions.

## Introduction

### Disruptive Mood Dysregulation Disorder

The Disruptive Mood Dysregulation Disorder (DMDD) has been included as a new diagnostic in the depressive disorder section of the Diagnostic and Statistical Manual of Mental Disorders, Fifth Edition (DSM-5) (1). It is characterized by persistent irritable mood and severe (i.e., out of proportion in intensity or duration) and frequent (i.e., three or more times per week) temper outbursts. The recurrent temper tantrums and persistent irritability/anger have been present for 12 months or longer, without free-symptom period for more than three months without all of the diagnostic symptoms throughout this period. The prevalence of DMDD ranges from 0.8% to 3.3% in the general population (2) and from 8% to 31% in outpatient samples (3).

This DMDD was operationalized on the basis the criteria for the syndrome of Severe Mood Dysregulation (SMD), a clinical entity developed to label youths misdiagnosed as having an early form of bipolar disorders (4). Carlson et al. (5) noted in a monograph of SMD children that these patients generally present impairments in multiple developmental domains, including cognitive processes, perceptual skills, and motor competencies. Over the last decade, several studies have confirmed that youths with DMDD present increased salience for emotional negative stimuli with impaired face-emotion labeling ability and a perceptual bias toward threatening faces compared to healthy children (6–9). What is less clear, however, is whether DMDD youths present difficulties for modulating nonvisual sensory perceptions such as touch, smell, taste, sound, body movement, or body position modalities. In a case series of 13 inpatient adolescents with SMD between 7 and 17, Guilé (10) found that all of them had both perceptive and motor difficulties as measured by a standardized motor assessment including the Movement Assessment Battery for Children-Second Edition (MABC-2), the Bender test, and the Concise Evaluation Scale for Children’s Handwriting (BHK). Participants scored particularly low in tasks measuring bodily perception (the Berges somatognosia test and the Goodenough–Harris Drawing Test). More recently, Iancu (11) conducted a chart review of 192 inpatient adolescents to compare the rate of learning disabilities in DMDD and in Major Depressive Disorder (MDD). He noted that adolescents with DMDD were twice as likely as those with MDD to have a developmental coordination disorder, with 43% of them having difficulties in visual–spatial perceptions as measured by the Developmental Test of Visual Perception, Second Edition (DTPV-2).

### Sensory Processing Difficulties and Psychopathology

Sensory processing is a neurological process that involves the perception of external and internal sensations by sensory receptors, the organization, and the interpretation of these sensations in order to effectively plan motor output and emotional responses (12). Planning the motor output and emotional responses requires deciding how to respond and regulate the intensity and duration of the reaction, which is referred to as modulation. Ultimately, adaptive modulation involves regulating the balance between habituation (i.e., when the sensory stimulus becomes more familiar, there is a decreased response) and sensitization (i.e., when the sensory stimulus is perceived as significant or threatening, an increased or maintained response occurs). Difficulty modulating sensory information such as touch, smell, taste, sight, sound, body movement, or body position can lead to abnormal patterns of sensory processing (12). Dunn’s sensory processing framework proposes an interaction between neurological threshold (amount of stimuli needed for the nervous system to notice or react to stimuli) and self-regulatory behavioral response (manner in which a person responds in relation to the threshold). Four processing patterns result from the intersection of these two continua: low registration (high thresholds with passive self-regulation), sensory seeking (high thresholds with active self-regulation), sensory sensitivity (low thresholds with passive self-regulation), and sensation avoiding (low thresholds with active self-regulation).

Problematic sensory processing patterns have primarily been described in autistic youths (13). It has thereafter been observed in children who present different developmental disorders, such as intellectual disability (14), fragile X syndrome (15), attention deficit disorder (ADD) (16), and learning disabilities (17, 18). Recently, researchers have expanded their attention showing that impaired sensory processing is associated with internalizing and externalizing behavior problems both in clinical (19) and general populations (20–27).

### Sensory Processing Difficulty and Emotional Dysregulation

Few studies have examined the specific association between emotional dysregulation and SPD. Cheng and Boggett-Carsjens (28) present the case of a 9-year-old boy with severe affect regulation problems and SPD. The child’s recurrent temper outbursts were finally connected to his feeling of being under continual sensory overload with a constant state of danger. The child’s mood lability, i.e., the inconsistent emotional and behavioral responses to his daily environment, was a consequence of the child’s sudden feeling of being overwhelmed by his sensory inputs when his threshold was reached.

Levitt (23) conducted a cross-sectional study in 47 school-aged children (from 7 to 14 years old) to examine the relation between parent-reported Short Sensory Profile, the Child Behavior Checklist/4–18 (CBCL), and the Emotion Regulation Checklist. She found that as sensory processing patterns became more problematic, emotional dysregulation increased. However, as noted by the author, it remains to be seen whether or not this relation exists in a clinical sample of youths with severe and impairing emotional dysregulation where the identification of SPD could represent a therapeutic opportunity. This study aims to answer this question, particularly in determining the frequencies and the pattern of abnormal sensory processing in help-seeking youths with DMDD.

### Aims

The first aim of this study was to determine the frequency of SPD in DMDD youths compared to clinical and nonclinical controls. It is hypothesized that a substantial part of youths with DMDD would show SPD as measured by parent-reported response on the Sensory Profile. This is based on studies showing positive correlations between sensory processing and emotional dysregulation (23, 28). We expected to find in our sample a rate of SPD in the DMDD group in the same range as in a study conducted in attention deficit disorder (ADD) patients, i.e., between 40% and 60% (16, 21).

The second aim of this study was to determine the types of SPD in DMDD youths. Based on typical clinical vignettes provided by Dunn et al. (29) and preliminary evidences (23, 28), we did not expect that DMDD youths would match only one of the specific patterns of SPD but rather present a mixed picture of abnormal patterns of sensory processing (e.g., low on scores associated with sensation avoiding and registration).

The third aim of this study was to determine the relation between SPD and emotional dysregulation using a dimensional score in the whole clinical sample. It is hypothesized that there will be a significant and positive relation between the subscores at the Sensory Profile and the scores at the Affective Lability Scale (ALS-54) and the DMDD severity scale.

The fourth aim of the study was to explore the association between SPD and other dimensions of psychopathology. We sought to confirm the association between parent-reported SPD and the CBCL externalized problem subscales observed in previous studies (20, 21, 23, 24, 26, 27). We will also examine the relation between SPD and other psychopathological dimensions in this clinical sample using an exploratory approach.

## Methods

### Participants

The participants were recruited in outpatient or inpatient facilities at the Child and Adolescent Psychopathology Department of the Amiens University Hospital. The main inclusion criteria were aged between 6 and 16 years and having been referred to one of the outpatient or inpatient units for emotional disturbances. In particular, the department is a regional resource center for suicidal behaviors, and most of the participants were addressed for suicidal ideation/attempts. Subjects with intellectual disability, autistic spectrum disorder, drug/alcohol abuse, or sleep/vigilance disorders at admission were not included. Generally, DMDD differs from the vast majority of youths referred to these units as emotional disturbances should have a chronic course with an onset prior the age of 10. To increase the homogeneity of the clinical control group, we excluded participants with chronic mood disturbance distinct from DMDD (bipolar disorder, n = 2; persistent depressive disorder, n = 1; schizophrenia and schizoaffective disorder, n = 1).

### Setting and Study Design

After the child/adolescent and his/her legal representative had provided their written, informed consent, the study questionnaires were administered. The informant-based diagnostic questionnaires were administered first [the Kiddie Schedule for Affective Disorders and Schizophrenia for School-Age Children–Present and Lifetime Version (K-SADS-PL), including the DMDD module]. Then, other psychopathological questionnaires and the Sensory Profile were administrated. A subgroup of our sample was included in another research involving the use of a wrist actimeter (30). The study’s procedure was approved by the local independent ethics committee (CPP Nord Ouest 2, Amiens, France).

### Measurement

#### Sensory Profile

The Sensory Profile, developed by Dunn, is a 125-item parent-based questionnaire that measures sensory processing abilities and provides an overview of their impact on functional performance in daily life of youths 3 to 15 years of age (31). The questionnaire is completed by caregivers. It encompassed different sections. (i) The Sensory Processing section is made up of six item categories that reflect particular types of sensory processing as part of daily life (e.g., auditory processing or touch processing). (ii) The Modulation section contains five item categories that reflect various combinations of input for use in daily life (e.g., modulation related to body position and movement or modulation of sensory input affecting emotional responses). (iii) The Behavioral and Emotional Responses section contains three item categories that reflect emotional and behavioral responses that might indicate a child’s sensory processing abilities (e.g., emotional/social responses).

Nine factors based on principal-components factor analysis have also been identified: sensory seeking, emotionally reactive, low endurance/tone, oral sensory sensitivity, inattention/distractibility, poor registration, sensory sensitivity, sedentary, and fine motor/perceptual. On the basis of these factors, children can also be classified as fitting into one of the four general sensory processing “quadrants”: sensation seeking, sensation avoiding, sensory sensitivity, and low registration (12). The quadrant with the lowest score is considered to characterize a child’s sensory processing profile.

#### Psychiatric Diagnosis

The K-SADS-PL, a semi-structured diagnosis interview, was administered by one of the investigating clinicians. The parents were the informants in the present study. The K-SADS-PL psychometric properties were estimated as excellent: inter-rater reliability: κ = 0.93; test–retest reliability: intraclass correlation = 0.74–0.90 (32). The additional K-SADS-PL module developed by Leibenluft and colleagues was used (33). Each item was endorsed as *present* or *absent* or *unknown*. The diagnostic algorithm follows the international guidelines (34). In particular, DMDD was endorsed only if participants positively matched criteria for duration criteria, cross-domain impairment, and age of onset. Exclusion criteria for bipolar disorder were applied. DMDD was not retained as an exclusion criterion for Oppositional Defiant Disorder to present the comorbidity rate with Disruptive Behavioral Disorders. The psychometric properties of this *ad hoc* diagnostic section for DMDD have been measured in another sample of 12–15-year-old outpatients (N = 192) (internal validity Cronbach’s α = 0.90, inter-rater reliability κ = 0.87) (35).

#### Psychopathological Dimensions

The ALS-54 is a 54-item measure developed to assess affect regulation (36). Questions, rated on a 4-point Likert scale, are coded from 0 (*not true*) to 3 (*true*). The total score is the mean of all item responses divided by the number of responses, thus ranging from 0 to 3, with a score closer to 3 indicating greater affective lability. The ALS-54 has been adapted for children aged 6–16 with parent-reported information, with excellent internal consistency and retest reliability (37).

The CBCL is a parent-report measure that assesses problematic behaviors a child may exhibit (38). Responses are given on a 3-point Likert scale, ranging from 0 (*not true*) to 2 (*very true or often true*). Scores are divided into eight subscales: anxious/depressed, withdrawn/depressed, somatic complaints, social problems, thought problems, attention problems, rule-breaking behavior, and aggressive behavior. Regarding reliability, (38) reported good to excellent test–retest reliabilities that ranged from 0.82 to 0.95, as well as acceptable to excellent internal consistency that ranged from 0.62 to 0.96.

The Beck Depression Inventory-Second Edition (BDI-II) is a 21-item self-report inventory (with each item rated from 0 to 3) measuring the severity of depression. It informs of the severity of depressive symptoms. The studies conducted in adolescent inpatients using this scale report good validity and internal consistency, Cronbach’s α > 0.90 (39).

### Statistical Analyses

Comparisons were conducted between participants diagnosed with DMDD (n = 30) and those not diagnosed with DMDD (n = 18). Since normal distribution was not confirmed for most variables, the nonparametric Mann–Whitney test was used to compare the DMDD and control scores for continuous variables, Fisher exact test for categorical variables.

The first aim of this study was to determine the frequency of SPD in DMDD youths compared to clinical case controls. SPD has been operationalized, in line with prior reports as follows: showing two standard deviations or below, i.e., “definite difference” in at least one of the nine sensory profile factors (21). Considering the exploratory approach of this study, we focused on SPD and we did not use the stringent criteria for Sensory Processing Disorder. Fisher exact test was used to compare the proportion of SPD in the group with DMDD versus those without DMDD.

The second aim of this study was to determine the types of SPD in DMDD youths. First, the Mann–Whitney comparison test was used to compare the mean scores at each subscale of the Sensory Profiles (six sensory processing sections, five modulations, three behaviors, nine factors, and four quadrants) between the youths in the DMDD group and those in the clinical control group. Second, the scores at each subscale of the Sensory Profiles were compared with raw scores for typically developing same-age children provided by the Sensory Profile manual (29). Standardized scores for the French population were derived from a community-based study conducted in 561 youths with stratification based on socioeconomic factors (29). The Z-test for two independent populations with known standard deviation was used, and Cohen’s *d* was performed to assess effect sizes (0.2, 0.5, and 0.8 correspond to small, medium, and large effects).

The third aim of this study was to determine the relation between SPD and emotional dysregulation. Spearman correlation, a nonparametric test, was performed to explore the relations between the nine sensory processing factors and two measures of emotional dysregulation, i.e., the total score at the ALS-54 and the DMDD severity score in the whole sample (both in patients with DMDD and in clinical case controls).

The fourth aim of the study was to explore the association between SPD and other psychopathological dimensions. Spearman correlations were conducted to measure the association between the nine sensory processing factors, the CBCL subscores, and the BDI-II in the whole sample (both in patients with DMDD and in clinical case controls).

## Results

The mean clinical and sociodemographic features of the subjects in the DMDD group and the clinical control group are detailed **Tables 1** and **2**.

**Table 1:** Sociodemographic and clinical feature of youths with DMDD and the clinical control group.

	DMDD (n = 30)	Clinical control group (n = 18)	p
**Socio-demographic and social characteristics**			
Gender, male, n (%)	26 (87%)	25 (72%)	0.168^†^
Age (y) (mean ± SE)	12.24 ± 0.47	13.67 ± 0.52	0.058^††^
**DSM-5 psychiatric disorders**			
MDD, n (%)	8 (27%)	4 (22%)	0.888^†^
Adjustment disorder with depressed mood, n (%)	1 (4%)	12 (60%)	**<0.001** ^†^
Anxiety disorders, n (%)	6 (20%)	6 (33%)	0.485^†^
Post-traumatic stress disorder, n (%)	2 (7%)	4 (22%)	0.251^†^
Attention deficit disorder, n (%)	9 (30%)	2 (11%)	0.359^†^
Disruptive behavioral disorders, n (%)	15 (50%)	6 (22%)	0.243^†^
Substance misuse, n (%)	4 (13%)	3 (17%)	0.924^†^
**Other clinical factors**			
Suicidal ideation (past or current), n (%)	16 (53%)	17 (94%)	**0.024^†^**
Suicidal attempt (past or current), n (%)	10 (33%)	11 (61%)	0.382^†^

^†^Fisher exact test.

^††^Mann–Whitney test.

DMDD, disruptive mood dysregulation disorder; DSM-5, Diagnostic and Statistical Manual of Mental Disorders, Fifth Edition; MDD, major depressive disorder.

**Table 2:** Mean clinical score for DMDD youths and the clinical control group.

	DMDD (n = 30)	Clinical control group (n = 18)	p
**Clinical scores**			
DMDD severity score (mean ± SE)	27.29 ± 1.78	2.77 ± 1.14	**<0.001** ^†^
ALS-54 (mean ± SE)	63.30 ± 4.60	66.94 ± 0.71	0.621^†^
BDI-II (mean ± SE)	23.93 ± 2.61	21.53 ± 2.85	0.743^†^
CBCL Internalized score (mean ± SE)	65.58 ± 1.23	65.81 ± 1.86	0.856^†^
CBCL Externalized score (mean ± SE)	73.33 ± 1.65	65.09 ± 2.24	**0.010^†^**
1- anxious/depressed subscale	71.96 ± 1.57	70.44 ± 2.68	0.586^†^
2- withdrawn/depressed subscale	66.15 ± 2.02	64.56 ± 1.82	0.584^†^
3- somatic complaints subscale	58.62 ± 1.46	62.44 ± 2.13	0.121^†^
4-thought problems subscale	68.04 ± 1.95	65.75 ± 2.36	0.281^†^
5- social problems subscale	68.85 ± 1.80	62.75 ± 1.94	**0.049** ^†^
6- attention problems subscale	67.91 ± 1.60	60.88 ± 2.17	**0.008** ^†^
7- rule-breaking behavior subscale	69.04 ± 1.69	64.44 ± 2.23	0.185^†^
8- aggressive behavior subscale	77.61 ± 2.00	65.75 ± 2.51	**0.001** ^†^

^†^Mann–Whitney test.

ALS-54, Affective Lability Scale; BDI-II, Beck Depression Inventory-Second Edition; CBCL, Child Behavior Checklist/4–18; DMDD, disruptive mood dysregulation disorder.

### Aim 1: Rate of SPD in DMDD Youths

Fifty-three percent of the youths in the DMDD group had SPD. The rate of SPD was not statistically different between those with DMDD and those in the clinical control group (respectively, 53% vs. 33%, p = 0.405). The frequency rate did not differ across groups using a broader definition of SPD, including those with either a definite or a probable difference in sensory processing abilities (DMDD 83% vs. clinical control group 72%, *p* = 0.287).

### Aim 2: Patterns of SPD in DMDD Youths

#### Q2a: DMDD vs. Clinical Control Group

Youths with DMDD did not statistically differ from the clinical control group with regard to the mean score of the sensory processing items and the mean score of the sensory modulation items (**Table 3**). In the section “Behavioral and Emotional Responses,” participants with DMDD had a score lower for the behavioral outcome of sensory processing items compared to those in the clinical control group, with respectively *M* = 17.3 vs. *M* = 21.4, *U* = 2.034, *p* = 0.042.

**Table 3:** Comparison of Sensory Profile section scores among disruptive mood dysregulation disorder (DMDD) youth and the clinical control group.

	DMDD (n = 30)		Clinical control group (n = 18)		Mann–Whitney test	
	M	SD	M	SD	U	p
**Sensory Processing (categories 1–6)**						
A. Auditory processing	29.30	1.40	31.2	1.53	0.855	0.393
B. Visual processing	35.78	1.01	37.13	1.50	0.895	0.371
C. Vestibular processing	46.00	1.47	49.07	1.12	1.126	0.260
D. Touch processing	71.77	2.68	77.33	3.04	1.382	0.167
E. Multisensory processing	26.42	1.06	28.20	0.93	1.086	0.278
F. Oral sensory processing	49.77	2.00	52.93	1.01	0.244	0.807
**Sensory Modulation (categories 7–11)**						
G. Sensory processing related to endurance/tone	38.73	1.39	40.93	1.00	0.302	0.763
H. Modulation related to body position and movement	40.96	1.49	43.8	1.60	0.965	0.334
I. Modulation to movement affecting activity level	21.50	0.92	21.73	1.10	0	0.999
J. Modulation of sensory input affecting emotional responses	16.12	0.64	15.27	0.55	-1.405	0.160
K. Modulation of visual input affecting emotion and activity level	14.69	0.55	16.13	0.74	1.486	0.137
**Behavioral and Emotional Responses (categories 12–14)**						
L. Emotional/social responses	45.81	2.09	51.47	2.61	1.733	0.830
M. Behavioral outcomes of sensory processing	17.31	1.20	21.40	0.74	2.034	**0.042**
N. Items indicating thresholds for responses	11.96	0.46	13.27	0.28	1.609	0.108
**Factors (9 factors)**						
1. Sensory seeking	59.42	3.02	67.20	2.84	1.436	0.151
2. Emotionally reactive	40.77	2.18	46.87	2.37	1.774	0.076
3. Low endurance/tone	38.73	1.39	40.93	1.00	0.302	0.763
4. Oral sensory sensitivity	38.12	1.81	39.67	1.15	-0.415	0.678
5. Inattention/distractibility	21.89	1.39	23.13	1.70	0.597	0.551
6. Poor registration	31.58	0.93	32.67	0.95	0.707	0.480
7. Sensory sensitivity	18.19	0.47	19.00	0.54	1.264	0.206
8. Sedentary	13.19	0.89	12.33	1.34	-0.612	0.540
9. Fine motor/perceptual	10.65	0.56	13	0.44	2.592	**0.009**

In the sensory processing factors section, youths with DMDD had a lower score for the factor 9: “Fine motor/perceptual behavior” compared to the control group (*M* = 10.7, *M* = 13, *U* = 2.592, *p* = 0.009). However, for the mean score for the eight other factors of the Sensory Profile, there was no statistical difference between the DMDD and clinical control group.

The most frequent sensory processing pattern reported in the DMDD youths was the sensation avoiding type (40%), followed by the low registration (27%) and the sensory sensitivity types (20%), and less frequently the sensation seeking type (10%). The low registration type was more frequent in the DMDD group compared to those with other emotional problems (*p* = 0.042), while no differences were observed for the other types.

#### Q2b: DMDD vs. Typically Developing Youths

DMDD patients scored higher compared to general population scores in almost all sensory processing, modulation, and behavioral/emotional response items (**Table 4**).

- For sensory processing categories, large effect sizes were observed for touch processing items (*d* = 1.02) and multisensory processing (*d* = 1.30), suggesting that DMDD youths may be specifically impaired in these domains. No difference was observed for oral sensory processing. Differences in other categories were small to moderate, suggesting more discrete difficulties (auditory processing, visual processing and vestibular processing).
- For sensory modulation, a large effect size was observed only for the category of modulation related to body position and movement (*d* = 1.38). No difference was noted in the category of modulation of sensory input affecting emotional responses. The effect sizes of the differences in other categories were small to moderate (sensory processing related to endurance, modulation to movement affecting activity level, modulation of visual input affecting emotional response and activity level).
- For behavioral and emotional responses, all three categories presented large effect sizes between youths with DMDD and expected scores in same-age general population: emotional/social responses (*d* = 1.86), behavioral outcomes of sensory processing (*d* = 1.71), and items indicating thresholds for responses (*d* = 1.03).
- For sensory profile factors, DMDD scored lower for items associated with a pattern of low registration: Factor 6: “poor registration” (*d* = 0.97), Factor 3: “low endurance tone” (*d* = 0.79), but not Factor 8: “sedentary” (*d* = 0.10). Youths with DMDD scored lower for items associated with a pattern of sensation avoiding: Factor 2: “emotionally reactive” (*d* = 1.09), but not Factor 8.

**Table 4:** Comparison of Sensory Profile section scores among disruptive mood dysregulation disorder (DMDD) youth and expected score in the general population.

		DMDD (n = 30)		Expected scores in the general population			Z-test for independent population			Cohen’s *d*	
		M	SD	M		SD	z-score		p		
**Sensory Processing (categories 1–6)**											
	A. Auditory processing	29.30	1.40		32.86	4.70		-4.149	**<0.001**		0.76
	B. Visual processing	35.78	1.01		38.10	4.26		-2.983	**0.003**		0.54
	C. Vestibular processing	46.00	1.47		48.23	6.01		-2.032	**0.042**		0.37
	D. Touch processing	71.77	2.68		80.06	8.16		-5.564	**<0.001**		1.02
	E. Multisensory processing	26.42	1.06		30.55	3.18		-7.114	**<0.001**		1.30
	F. Oral sensory processing	49.77	2.00		51.68	7.58		-1.380	0.168		0.25
**Sensory Modulation (categories 7–11)**											
	G. Sensory processing related to endurance/tone	38.73	1.39		41.88	3.97		-4.346	**<0.001**		0.79
	H. Modulation related to body position and movement	40.96	1.49		46.24	3.82		-7.571	**<0.001**		1.38
	I. Modulation to movement affecting activity level	21.50	0.92		23.08	3.76		-2.301	**<0.001**		0.42
	J. Modulation of sensory input affecting emotional responses	16.12	0.64		17.18	2.81		-2.066	0.038		0.38
	K. Modulation of visual input affecting emotional responses and activity level	14.69	0.55		16.77	3.03		-3.760	**<0.001**		0.69
**Behavioral and Emotional Responses (categories 12–14)**											
	L. Emotional/social responses	45.81	2.09		66.04	10.86		-10.203	**<0.001**		1.86
	M. Behavioral outcomes of sensory processing	17.31	1.20		24.47	4.18		-9.383	**<0.001**		1.71
	N. Items indicating thresholds for responses	11.96	0.46		13.58	1.57		-5.657	**<0.001**		1.03
**Factors (9 factors)**											
	1. Sensory seeking	59.42	3.02		69.78	9.07		-6.256	**<0.001**		1.14
	2. Emotionally reactive	40.77	2.18		61.80	11.02		-10.452	**<0.001**		1.09
	3. Low endurance/tone	38.73	1.39		41.88	3.97		-4.346	**<0.001**		0.79
	4. Oral sensory sensitivity	38.12	1.81		38.87	6.49		-0.633	0.529		0.12
	5. Inattention/distractibility	21.89	1.39		26.87	4.59		-5.942	**<0.001**		1.08
	6. Poor registration	31.58	0.93		35.85	4.38		-5.340	**<0.001**		0.97
	7. Sensory sensitivity	18.19	0.47		18.38	2.14		-0.486	0.624		0.09
	8. Sedentary	13.19	0.89		12.84	3.41		0.562	0.575		0.10
	9. Fine motor/perceptual	10.65	0.56		13.11	2.27		-5.937	**<0.001**		1.08

### Aim 3: Correlation Between SPD and Emotional Dysregulaton

The DMDD severity score was significantly correlated with the following factors: sensory seeking, emotionally reactive, poor registration, and fine motor/perceptual (**Table 5**). The correlation between the ALS-54 total score and the sensory processing factors was not significant.

**Table 5:** Correlation between Sensory Profile factors and emotional dysregulation in all clinical subjects.

	Sensory Profile Factors								
	1 Sensory seeking	2 Emotionally reactive	3 Low endurance/tone	4 Oral sensory sensitivity	5 Inattention/distractibility	6 Poor registration	7 Sensory sensitivity	8 Sedentary	9 Fine motor/perceptual
DMDD severity score	rs = -0.31	rs = -0.40	Ns	ns	ns	rs = -0.37	ns	ns	rs = -0.36
ALS-54	ns	Ns	Ns	ns	ns	ns	ns	ns	ns

ALS-54, Affective Lability Scale; DMDD, disruptive mood dysregulation disorder.

### Aim 4: Correlation Between SPD and Other Psychopathological Dimensions

Negative correlations were found between the CBCL externalized score and different factors of sensory processing factors: sensory seeking, emotionally reactive, inattention/distractibility, poor registration, sensory sensitivity, and fine motor/perceptual (**Table 6**). The CBCL internalized score was only associated with a low endurance factor and poor registration factor. The BDI-II was not associated with any sensory profile factors.

**Table 6:** Correlation between Sensory Profile factors and other psychopathological dimensions in all clinical subjects.

	Sensory Profile Factors								
	1 Sensory seeking	2 Emotionally reactive	3 Low endurance/tone	4 Oral sensory sensitivity	5 Inattention/distractibility	6 Poor registration	7 Sensory sensitivity	8 Sedentary	9 Fine motor/perceptual
BDI-II	ns	ns	ns	ns	ns	ns	ns	ns	ns
CBCL Internalized score	ns	ns	rs = -0.40	ns	ns	rs = -0.34	ns	ns	ns
CBCL Externalized score	rs = -0.50	rs = -0.55	ns	ns	rs = -0.44	rs = -0.38	rs = 0.35	ns	rs = -0.40
1- anxious/depressed subscale	ns	ns	rs = - 0.32	ns	ns	ns	ns	ns	ns
2- withdrawn/depressed subscale	ns	ns	ns	ns	ns	rs = -0.64	ns	ns	ns
3- somatic complains subscale	ns	ns	ns	ns	ns	ns	ns	ns	rs = 0.45
4-thought problems subscale	ns	rs = -0.63	ns	ns	rs = -0.35	ns	ns	ns	ns
5- social problems subscale	rs = -0.43	rs = -0.45	rs = -0.39	ns	rs = -0.47	rs = -0.45	ns	ns	rs = -0.36
6- attention problems subscale	rs = -0.55	rs = -0.54	ns	ns	rs = -0.61	rs = -0.56	ns	ns	rs = -0.39
7- rule-breaking behavior subscale	rs = -0.42	ns	ns	ns	ns	ns	ns	ns	ns
8- aggressive behavior subscale	rs = -0.51	rs = -0.64	ns	ns	rs = -0.47	rs = -0.38	ns	rs = 0.36	rs = -0.46

BDI-II, Beck Depression Inventory-Second Edition; CBCL, Child Behavior Checklist/4–18.

## Discussion

### Interpretation

This study aims to determine the frequency and the patterns of SPD in a clinical sample of youths with DMDD compared to controls.

#### Q1: Rate of SPD in DMDD Youths

Using the parent-reported Dunn Sensory Profile, we found that 53% of the youths in the DMDD group had SPD. This frequency was in the same range as reported in a clinical sample of children with ADD patients (40%–60%) (16, 21). The high rate of SPD in youths with DMDD could be attributable to a number of factors that we develop here.

As a preliminary point, it is important to make sure that the high co-occurrence rate is not attributable to overlapping symptoms between SPD and DMDD, especially since the sensory processing problems lack a precise operational definition (25). For example, existing descriptions of SPD encompass a wide array of maladaptive emotional and behavioral responses, e.g., aggression, irritability, moodiness (12) which are part of the definition of DMDD. As both SPD and DMDD increase the likelihood of being admitted to an outpatient psychiatric facility, the rate of comorbidity between the two conditions reported here may be influenced by Berkson’s bias (40).

A first hypothesis to explain the high co-occurrence rate between SPD and DMDD is that the two clinical entities share at least partly common etiological mechanisms that make them more likely to occur simultaneously. This assumption is implicitly assumed by Ayres (41) who postulated that self-regulation skills (including emotional regulation) are an end product of sensory integration. This would also be in line with the general assumption that affect regulation is a high-order cognitive function progressively acquired through development *via* the interplay between early sensory, motor, and perceptual abilities (42). Empirical data have shown that SPD and chronic affective dysregulation might share common risk factors, such as pregnancy/birth factors (i.e., prematurity, low birth weight, obstetrical complications, or prenatal exposure to certain medications or alcohol) (43–45). Moreover, symptoms of SPD tend to be negatively correlated with regulatory aspects of temperament frequently reported in infants with poor affective control, such as low inhibitory control and soothe ability, and more negative effects such as anger, sadness, and fear (46–48).

An abnormal sensitivity to contextual and explicit threat cues has been regarded as a pathological process underpinning DMDD symptoms (49, 50). During facial emotion recognition tasks, youths with DMDD tend to have attentional biases to threat-relevant stimuli and threat-based appraisals of ambiguous stimuli (51). One possible hypothesis could be that youths with DMDD present a failure to habituate to repeated emotionally-valence sensory stimulation. A slightly distinct but complementary view would be that SPD may be a phenomenological characteristic of a child with severe affective dysregulation, as mentioned by Conelea et al. (19). Youths with early SPD, in particular from interoceptive system, would experince inconsistent and chaotic perceptions from their own body. Following Barrett’s (52) view, youths having more difficulties to categorize inner perceptions would ultimately have difficulties to label them with an emotional concept. As sensory modulation of inner perceptions is seen as a crucial step for emotion perceptions (53), according to this view, a child with SPD would have more difficulties to map elaborate mental representation of their own feelings, leading to low emotional awareness and ultimately poor emotional control.

Finally, it can also be speculated that youths with SPD may have fewer opportunities for peer relations and to then develop effective socio-emotional abilities. SPD may limit a child’s social and academic participation with opportunities to learn self-regulation strategies (12, 54). One may also ask how the child’s difficulties to integrate multimodal sensations, including body position, could interfere with his early interactions and parental bonding involved at different steps of the development of emotional regulation (55).

#### Q2: Comparison Between DMDD, Clinical Control Group, and Typically Developing Youths

The participants with DMDD show a significant difference on almost all items of the Sensory Profile when compared to typically developing children. However, the Sensory Profile was found to discriminate best between the participants with DMDD and those in the clinical control group with regard to the category M “Behavioral outcomes of sensory processing” and the Factor 9 “Fine motor/perceptual behavior.” The raw scores for category M were found to be lower in the sample of youths with DMDD. This may be explained by the fact that DMDD youths who experience difficulties in sensory processing will find it more difficult to meet the demands set by the environment which will then result in more emotional responses such as frustration and emotional outbursts. The significantly lower scores found in the sample of youths with DMDD for Factor 9 is also congruent with the reported characteristics of children with DMDD. Iancu (11) found that adolescents with DMDD reported a higher rate for fine motor problems compared to adolescents with major depressive disorder. Interestingly, difficulties to modulate body position and movement dimensions seem particularly impaired in the sample of DMDD youths. According to the data summarized in **Table 3**, youths with DMDD did not match any one specific pattern of SPD, supporting our *a priori* hypothesis that DMDD patients present a mixed picture of abnormal patterns of sensory processing.

#### Q3 and Q4: Correlation Between SPD, Emotional Dysregulation, and Other Psychopathological Dimensions

As SPD and irritability have both been described in children as dimensional problems (3, 12), we found it valuable to explore the relation between the two entities using a dimensional approach within our clinical sample. The data from this research found a significant correlation between the DMDD severity score and four factors of the SPD, but not with the ALS-54. The discrepancies between the two findings may result from the difference in what is measured by the two scales. The DMDD severity score measures how severe and impairing DMDD symptoms can be, focusing only on irritability. By contrast, the ALS-54 assesses various domains of affective regulation, whatever the valence of the emotion (e.g., positive for elation or negative for depression, anxiety, and anger). Moreover, the duration of emotional disturbance (episodic vs. chronic) is not taken into account by the ALS-54. That may explain why, paradoxically, DMDD youths have a lower score at the ALS-54 compared to clinical controls. Our data suggest that the relation between emotional dysregulation and SPD, if confirmed, is not limited to youths with chronic irritability.

In line with prior studies, we confirmed the relation between SPD and externalizing problems (20–27) as well as with internalizing behaviors (19). However, no significant relations were found between SPD and a measure of depressive symptoms (BDI-II). Such findings could indirectly support the role of psychopathological dimensions associated with internalized symptoms but distinct from depression, such as anxiety, in SPD youths. Conelea et al. (19) found a high rate of sensory over-responsivity symptom in a clinical sample of children with anxiety symptoms, 93% of whom were bothered by at least one tactile or auditory sensation.

### Strengths and Limitations

The current study has several limitations. The first limitation refers to the measurement issue. The Sensory Profile is a parent-reported measure. Including multi-informant ratings of SPD and psychopathology may help further elucidate the relationship between these constructs. A typical score was obtained from manual information based on a national data sample that is several years old and may not be a good matched sample. Moreover, in our sample, eight subjects were adolescents above 15. However, rerunning an analysis excluding these eight subjects did not alter the main findings of this study.

The second notable limitation includes the nature of the control sample. As mentioned above, using a control group enriched in youths with acute emotional disturbance (including anxiety and depressive symptoms) makes difficult to determine whether the high rate of SPD in DMDD youths is truly specific to their emotional dysregulation problem. Further studies should use different clinical control groups such as predominant emotional disturbance, predominant behavioral disorders, and combined problems.

The third limitation is linked to the measures of emotional dysregulation. Considering the high rate of comorbidity of DMDD with other childhood psychiatric disorders (3), it would be worthwhile to determine in further studies which clinical dimensions in DMDD youths explain the high rate of SPD, in particular the contribution of irritability in addition to anxiety symptoms, attention deficit, and aggressive behavior. In this study, unfortunately, the small size of the sample precludes us from performing a multivariate analysis to determine the specific role of these psychopathological dimensions.

The fourth limitation concerns the low size of the sample resulting in possible sampling bias and lack of statistical power. As mentioned above, this sample reflects adolescents referred in a tertiary university care center mostly for suicidal behaviors. How these findings apply to adolescent with less severe symptoms remains to be studied. In addition, the nonsignificant difference between the DMDD and the clinical control groups is difficult to interpret considering the possible lack of statistical power.

## Conclusion

The results of this study indicate that, as a group, youths with DMDD have significantly more SPD when compared to typical youths but not compared to youths with other emotional disturbances. Further analysis of the data revealed that although youths with DMDD exhibit the full range of sensory processing, they may be particularly impaired in the modulation of perception from body position and movement. If confirmed, this finding shows that SPD could be an important factor to consider in youths with DMDD for both comprehensive assessment and when designing interventions to support these patients’ symptoms and family difficulties.

## Data Availability Statement

The datasets generated for this study are available on request to the corresponding author.

## Ethics Statement

The studies involving human participants were reviewed and approved by CPP Nord Ouest 2, Amiens, France. Written informed consent to participate in this study was provided by the participants’ legal guardian/next of kin.

## Author Contributions

XB, DC, and JG made substantial contributions to the conception and design of the work. XB, VB, HL, DC, and JG made substantial contributions to the acquisition, analysis, or interpretation of data. XB, LD, and JG drafted the work or revised it critically for important intellectual content. XB, VB, HL, LD, and JG gave final approval of the version to be published. XB, VB, HL, LD, DC, and JG agreed to be accountable for all aspects of the work to ensure that questions related to the accuracy or integrity of any part of the work are appropriately investigated and resolved.

## Funding

The present research was funded by grant AOL11 No. 2012- A00925-38 from Amiens University Hospital.

## Conflict of Interest

The authors declare that the research was conducted in the absence of any commercial or financial relationships that could be construed as a potential conflict of interest.
